# Database of lower limb kinematics and electromyography during gait-related activities in able-bodied subjects

**DOI:** 10.1038/s41597-023-02341-6

**Published:** 2023-07-14

**Authors:** Robert V. Schulte, Erik C. Prinsen, Leendert Schaake, Robert P. G. Paassen, Marijke Zondag, Eline S. van Staveren, Mannes Poel, Jaap H. Buurke

**Affiliations:** 1 Roessingh Research & Development, Enschede, 7522AH The Netherlands; 2 University of Twente, Department of Biomedical Signals & Systems, Enschede, 7522NB The Netherlands; 3 University of Twente, Department of Biomechanical Engineering, Enschede, 7522NB The Netherlands; 4 University of Twente, Department of Data Management & Biometrics, Enschede, 7522NB The Netherlands

**Keywords:** Biomedical engineering, Machine learning

## Abstract

This data descriptor describes the Roessingh Research & Development-MyLeg database for activity prediction (MyPredict), containing three data sets. These data sets contain data from 55 able-bodied subjects, mean age 24 ± 2 years, measured in 85 measurement sessions. Measurement sessions consisted of trials containing sitting, standing, overground walking, stair ascent, stair descent, ramp ascent, ramp descent, walking on uneven terrain and walking in simulated confined spaces. Subjects were measured using eight inertial measurement units in combination with different types of sEMG. Recorded kinematics consisted of joint angles, sensor accelerations, angular velocity, orientation and virtual marker positions. sEMG was recorded using bipolar sEMG, multi-array sEMG or a combination of both. All data showed excellent correlation with other online available data sets. The data reported in this descriptor forms a solid basis for research into myoelectric pattern recognition, myoelectric control development and electromyography to be used in data-driven applications.

## Background & Summary

Human motor intent recognition based on surface electromyography (sEMG) could provide a more intuitive control in applications such as prostheses, exoskeletons or wheelchairs. sEMG is a non-invasive technique and therefore well suited to realize intent recognition. However, analysis of sEMG is complex due to its stochastic nature. Robustness of a myoelectric system is important, but due to limiting factors in sEMG such as muscle fatigue, electrode shift and inter-subject variability, this is difficult to realize^1^. To capture this variability, large amounts of data are necessary. The sharing and availability of data is also necessary if the field moves towards more big data applications, such as deep learning. Deep learning shows the promise of reaching better performing and more general applicable algorithms compared to more traditional methods developed on small data sets. The downside of these data-driven approaches is that large amounts of data are necessary. Therefore, it is important for the myoelectric control research community to share sEMG related data^1^.

sEMG collection has become easier over time and more and more data sets containing sEMG are made publicly available, especially in the upper extremity. In the upper extremity sEMG control research has been around for decades and myoelectric control is making its way slowly to the lower limb^2,3^. The limited amount of research into lower limb myoelectric control is one of the main reasons why databases containing sEMG and kinematics of the lower limb are limited. Fortunately, in recent years the amount of research into myoelectric control grows and more data becomes available^3–9^. For instance Hu *et al*.^6^ presented a benchmark data set containing sEMG and kinematics measured with wearables collected during free transitioning of various gait-related activities in 10 able-bodied subjects. Camargo *et al*.^7^ measured 22 able-bodied subjects during gait-related activities such as walking, stair climbing and ramp walking. Lencioni *et al*.^9^ created a database containing 50 able-bodied subjects, performing gait-related activities, such as walking, stair walking and walking on toes and heels. These studies provide valuable data sets for the research community to use, to gain more insight into human mechanics of the lower limb and also move the field towards data-driven applications.

Important to note is that in most of these data sets the subjects did not transition freely from one activity to the next, except for the database by Hu *et al*.^6^. Data of these free transitions are required for a more realistic data set, closely matching a daily life setting. Realistic data is necessary for developing methods that can be used in home environments and contain variability that can be expected during daily use. Next to that, no database contained multi-array sEMG, although these grids of electrodes could give more meaningful insight into human motor function and enables more complex analysis methods^1,10^.

In this work we present the Roessingh Research & Development-MyLeg database for activity prediction (MyPredict). The general aim of this database is to promote research in data-driven intent recognition strategies and activity prediction strategies in the lower-limb using electromyography and to promote research and development in the area of multi-array sEMG in the lower limb. The database contains three data sets, each containing kinematics and sEMG from able-bodied subjects. In total 55 subjects participated over 85 measurement sessions. Each data set contained a different sEMG measuring protocol containing either traditional bipolar sEMG or multi-array sEMG or a combination of both. In these data sets the subjects transitioned freely from one activity to the next, providing challenging data sets for activity recognition and providing the possibility to investigate human kinematics and sEMG during gait-related activities. It should be kept in mind that this database consists of young able-bodied individuals. However, this database might provide a meaningful starting point for analyses into for instance activity recognition during gait-related activities and analyses into transition periods between two activities. Parts of these data sets were used in earlier work^11,12^, but this is the first time the data becomes publicly available.

## Methods

### Materials

In each data set lower body kinematics were collected using an MVN Link suit (Xsens, Enschede, The Netherlands), which uses eight inertial measurement units (IMUs) to reconstruct lower body movement at 240 Hz. IMUs were placed on the feet, lower legs, upper legs, pelvis and sternum. Details on the exact anatomical placement of IMUs are available in the documentation provided by Xsens^13^. Recorded kinematics were 3D acceleration and angular velocity per sensor, 3D reconstructed lumbar, pelvic, hip, knee and ankle angle and 3D virtual marker positions. The acceleration in the data set is so-called sensor-free acceleration, which means that the gravity component is subtracted. sEMG was recorded using three measurement systems and four different configurations were used, which are outlined below. Bipolar sEMG placement was done according to SENIAM guidelines^14^. An overview of the used measurement systems per dataset is shown in Table 1. The sensor locations are indicated in Fig. 2. Kinematics collected by the MVN link suit and the sEMG collected by the measurement systems were time synchronized and resampled to 1000 Hz. Synchronization between various measurement systems was performed using a validated synchronization method based on acceleration cross-correlation^15^. Marker positions were resampled to 100 Hz to reduce file size.

**Table 1:** Overview of MyPredict 1–3. Top: used measurement systems and measurement set-ups, bottom: subject characteristics.

	MyPredict 1	MyPredict 2	MyPredict 3
Kinematics	Xsens, 240 Hz	Xsens, 240 Hz	Xsens, 240 Hz
Bipolar sEMG	Delsys Trigno, 1000 Hz	—	Cometa Wave, 2000Hz
Bipolar muscles	Gmax, RF, VL, BF,	—	Gmax, RF, VL, BF,
	ST, TA, GM, Gmed		ST, TA, GM, AM
Multi-array sEMG	—	Sessantaquattro, 2000Hz	Sessantaquattro, 2000Hz
Grid	—	front/back 4 × 8 ied 10 mm	4 × 16, ied 20 mm
Subjects	10	35	10
- No. moments	1	1	4
- Sex (male/female)	7/3	14/21	4/6
- Age (years)	24 ± 2	23 ± 2	24 ± 2
- Weight (kg)	77 ± 10	73 ± 11	71 ± 9
- Height (cm)	183 ± 9	174 ± 9	174 ± 6

The used software for Xsens recordings was MVN Analyze v2019^13^.

#### MyPredict 1

MyPredict 1 only contained bipolar sEMG. Bipolar sEMG was collected for MyPredict 1 using Trigno electrodes (Delsys, Boston, US) at a sample frequency of 1000 Hz. The measured muscles were the gluteus maximus (Gmax), rectus femoris (RF), vastus lateralis (VL), biceps femoris (BF), semitendinosus (ST), tibialis anterior (TA) and gastrocnemius medialis (GM) and the gluteus medius (Gmed). sEMG acquisition software was a custom MATLAB script using MATLAB 2017a^16^.

#### MyPredict 2

**Fig. 1:**
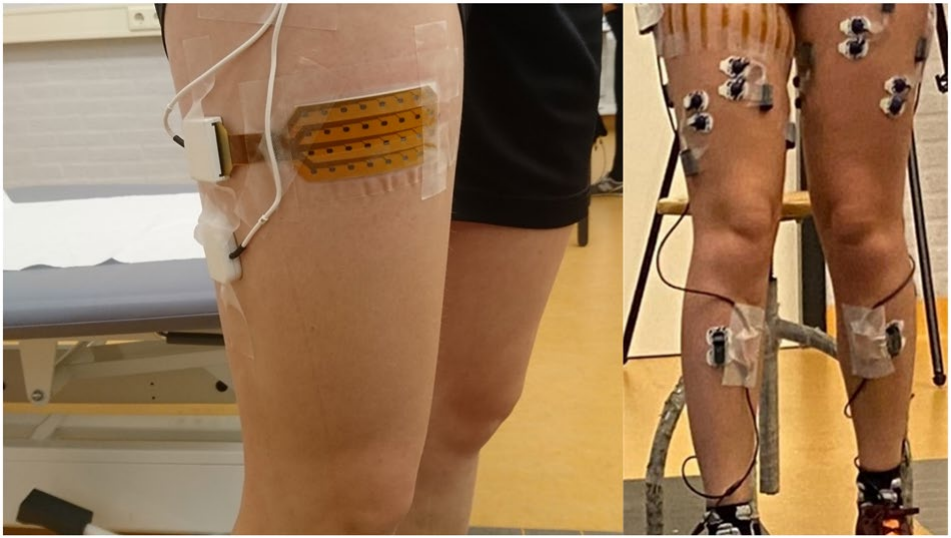
Multi-array sEMG grids used in MyPredict 2 (**A**) MyPredict 3 (**B**). The bipolar sEMG and Xsens IMU are also visible in (**B**). MyPredict 1 did not contain multi-array sEMG.

MyPredict 2 only contained multi-array sEMG. Multi-array sEMG signals for MyPredict 2 were recorded using the Sessantaquattro (Bioelettronica, Turin, Italy) with two sEMG grids (GR10MM0804) in a 4 × 8 configuration and an inter-electrode distance of 10 mm at a sample frequency of 2000Hz, see Fig. 1A. The software for multi-array sEMG acquisition was OTBiolab+ v1^17^.

**Fig. 2:**
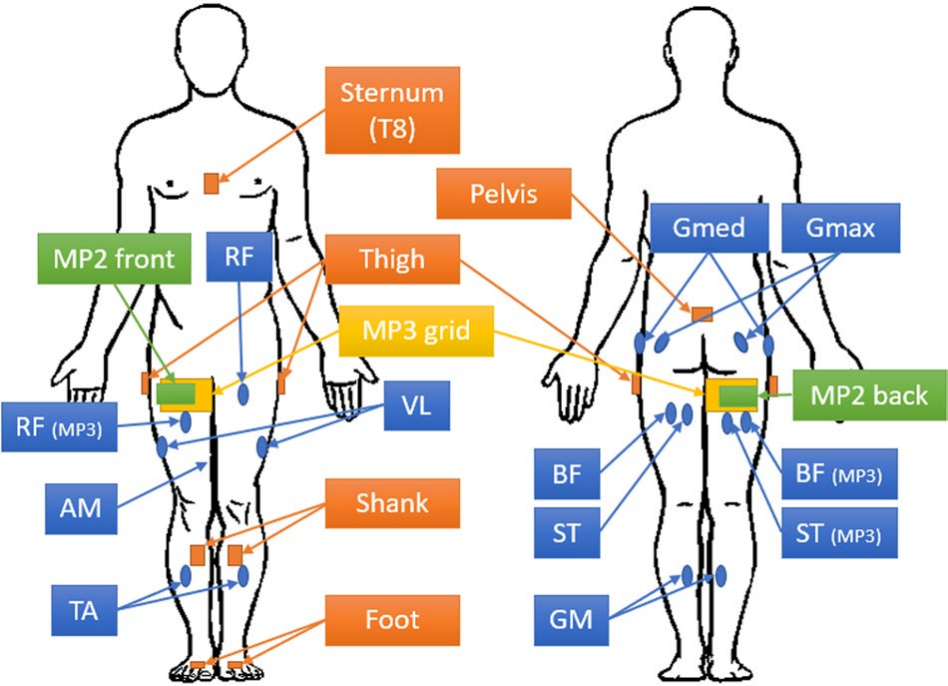
Sensor locations of the different modalities. Xsens IMUs are indicated in orange, the bipolar sEMG (MP1 and MP3) are indicated in blue. Note that for MP3 the bipolar sEMG locations differed slightly on the right leg, as the MP3 sEMG multi-array grid (indicated in yellow) was positioned there as well. The multi-array grids front and back (MP2) are indicated in green.

#### MyPredict 3

MyPredict 3 contained multi-array sEMG and bipolar sEMG. Bipolar sEMG for MyPredict 3 was acquired using the Wave electrodes (Cometa Systems, Bareggio, Italy) at a sampling frequency of 2000 Hz. The measured muscles using bipolar sEMG were the Gmax, RF, VL, BF, ST, TA, GM and the adductor magnus (AM). Multi-array sEMG was recorded with the Sessantaquattro (Bioelettronica, Turin, Italy) at a sampling frequency of 2000Hz and a custom-made grid of 4 × 16 electrodes with an interelectrode distance of approximately 20 mm. The grid covered the upper leg, spanning from the vastus lateralis to the aductor magnus and ending at the biceps femoris at the back, see also Fig. 1B. The software for multi-array sEMG acquisition was OTBiolab+ v1^17^ and the software for bipolar sEMG acquisition was sEMG and Motion tools v7^18^.

### Protocols

#### Ethical statement

The data collection protocols were reviewed and approved by Medical research Ethics Committees United (MEC-U) Nieuwegein, the Netherlands, with trial number NL67247.044.18. The participants provided their written informed consent before inclusion in the studies.

#### MyPredict 1

**Fig. 3:**
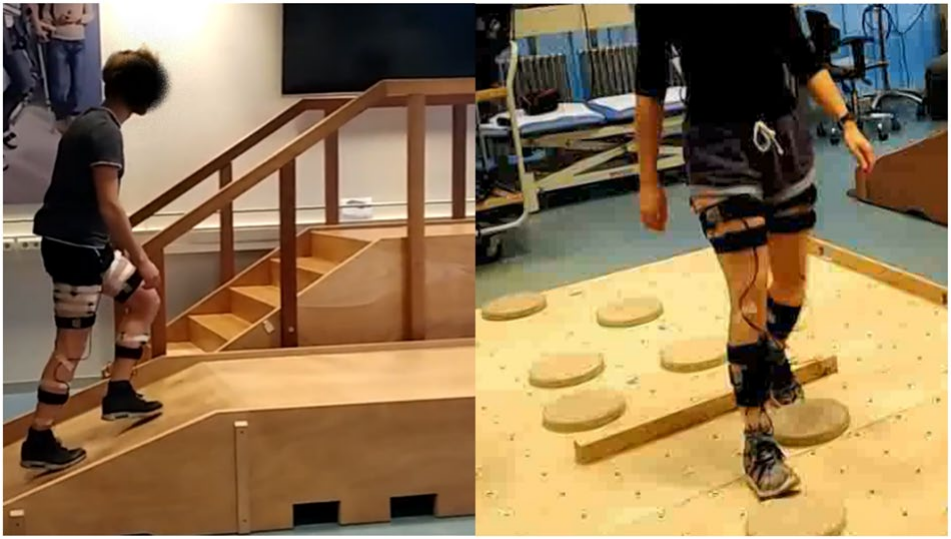
Obstacles used in MyPredict 1. (**A**) Ramps and stairs (image taken from Schulte *et al*.^11^) and (**B**) uneven terrain.

10 able-bodied subjects (sex: 7 m, 3 f; age: 24 ± 2 years; weight: 77 ± 10 kg; height: 183 ± 9 cm) participated in this part of the study. Measurements were conducted at the Wearable Robotics Lab of the University of Twente, using obstacles constructed for the Cybathlon by the Department of Biomechanical Engineering. Obstacles used were the stairs (rise 17 cm, run 28 cm), ramp with two different slopes (15 and 20 degrees) and uneven terrain consisting of stepping stones on a surface, see also Fig. 3. Forty trials were conducted per subject. A trial consisted of sitting, standing, walking, stair ascent, walking, stair descent, walking, ramp ascent, walking, ramp descent, walking, walking on uneven terrain, walking in confined spaces, walking, standing and sitting. Confined spaces consisted of the subject taking small steps in all directions, i.e. forwards, backwards, sidesteps to left or right, diagonally forwards, backwards to either left or right. Subjects walked at their own preferred speed and after ten trials a small break was administered to avoid fatigue and check sensor placement. Each trial had a duration of around 1.5 minutes. Total measurement time including subject preparation, sensor placement and calibration was around two hours.

#### MyPredict 2

35 able-bodied subjects (sex: 14 m, 21 f; age: 23 ± 2 years; weight: 73 ± 11 kg; height: 179 ± 9 cm) participated in this part of the study. To have more life-like data, measurements were conducted partly in a lab, but also outside. All measurements took place in and around Roessingh Research & Development, Enschede, The Netherlands. Each subject performed five types of trials in the same order: [noitemsep,topsep = 0 pt]

**Fig. 4:**
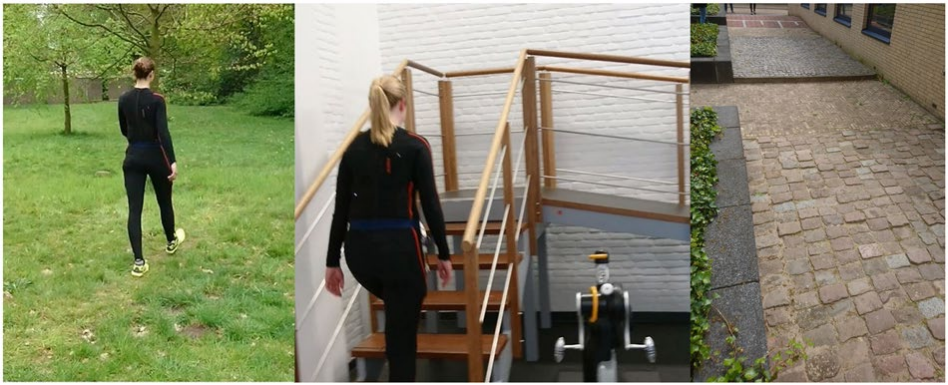
Some of the obstacles used in MyPredict 2. (**A**) Grass of the uneven terrain trial, (**B**) stairs/ramp combination and (**C**) Uneven terrain II. For uneven terrain II the trial started at the speed bump (top of the image), hereafter two types of cobblestones had to be crossed.
- **Stairs** Subject sat on a stair, stood up, walked to the stairs, ascended two flights of stairs, one consisting of eleven steps, the other of nine steps. Hereafter the subject stood still, turned around and descended the stairs, walked to the chair and sat down again.
- **Ramp** The trial started with ascending a staircase with seven steps, reaching a plateau and descending a ramp (10 degrees) which continued into a steeper ramp (15 degrees) after three meters. The subject stood still at the end of the ramp, turned around and ascended the ramp. Hereafter the subject descended on the stairs and turned around to start the trial again. See also Fig. 4B.
- **Uneven terrain II** This path consisted of uneven terrains found in the street. First the subject needed to come over a speed bump and hereafter walk on level ground towards the first type of cobblestones. This terrain consisted of small square stones which were slightly uneven. The subject crossed these cobblestones and walked onto the cobblestones consisting of unevenly laid Belgian blocks. After passing these cobblestones, the subject turned around and walked back over all types of terrains and repeated the trial. See also Fig. 4C.
- **Confined spaces** The subject lay on a bed, stood up and walked towards confined spaces set-up. The subject took small steps in all directions, i.e. forwards, backwards, sidesteps to left or right, diagonally forwards, backwards to either left or right.

- **Uneven terrain I** A trial consisted of sitting on a bench, standing up, walking on level ground, walking on grass, standing still and walking back and sitting down. See also Fig. 4A.

Between the trials the subject walked to each location and this data were recorded as well. Each trial was conducted ten times, with a total measurement time of around two hours, including subject preparation, sensor placement and calibration.

#### MyPredict 3

10 able-bodied subjects (sex: 4 m, 6 f; age: 24 ± 2 years; weight: 71 ± 9 kg; height: 174 ± 6 cm) participated in this part of the study. Data were collected at Roessingh Research & Development, Enschede, the Netherlands. Each subject was measured four times: three measurements were conducted on three subsequent days on day 1, 2 and 3 and the last measurement was three days later on day 7. The subjects were measured during the same time slot on each day. Each measurement included the same activities. Before each measurement the maximal voluntary contraction of each muscle was measured to be used for sEMG normalization.

**Fig. 5:**
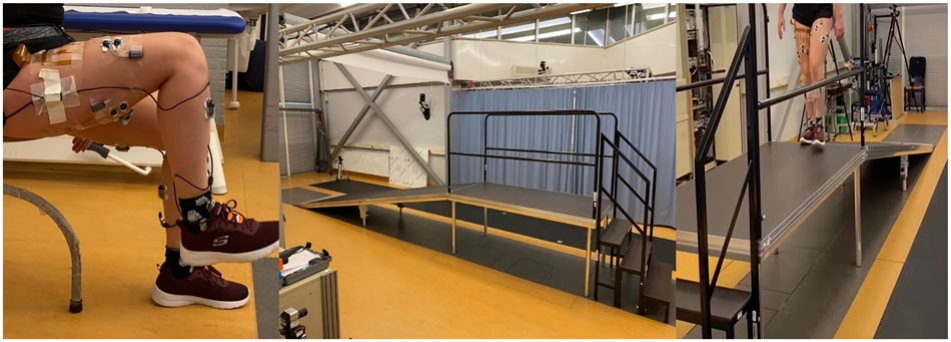
Measurement set-up of MyPredict 3. Trials started with non-weight bearing activities where the subject was seated on a stool (**A**) (taken from Schulte *et al*.^12^), hereafter the subject had to cross the stair/ramp combination from both sides, shown in (**B**) without subject and in (**C**) with subject.

The subjects were asked to perform a circuit of activities, including level-ground walking, stair ascent/descent (rise 20 cm, run 20 cm), ramp ascent/descent (10 degrees), sit-stand motions and non-weight-bearing activities on a stool. The subject had to sit on a stool and lift one leg off the ground (knee approximately 90 degrees). Then, the subject had to fully extend its knee while keeping its foot perpendicular to its lower leg. After, the subject performed maximal plantar flexion of the ankle, followed by maximal dorsiflexion. The knee was then brought back to a knee angle of approximately 90 degrees. Then, only knee extension and flexion needed to be performed. Lastly, only ankle plantar- and dorsiflexion needed to be performed while keeping the knee angle at 90 degrees. After, the foot was set down on the ground and the routine was repeated with the other leg. See also Fig. 5A. Hereafter the subject stood up, walked, ascended the stairs, walked, descended the ramp, walked, turned around, walked back to the ramp, ascended the ramp, walked, descended the stairs, walked and sat down again, see also Fig. 5B and 5C. This circuit was performed twenty times. Then, the routine was slightly changed for another twenty circuits: the subject had to first perform ankle plantar- and dorsiflexion, then the combination of both knee and ankle, and finish off with only knee extension and flexion. Remaining activities did not change in order. Total measurement time including subject preparation, sensor placement and calibration was around three hours per measurement day.

#### Maximum voluntary contraction for sEMG normalization

To normalize sEMG we recorded a maximum voluntary contraction (MVC) as well, which is based on the recommendations by Rutherford *et al*.^19^. The main difference is that we performed the contractions while standing instead of sitting. These MVCs were recorded for the bipolar sEMG of MyPredict 1 and MyPredict 3. No MVCs were recorded during the MyPredict 2 measurements. During the MVCs the subject was standing upright, using a wall or pole for balance and was asked to perform the following exercises, for a duration of five seconds:

- **RF-VL**: The hip and knee were flexed to approximately 90 degrees. The observer placed its hands on the anterior side of the lower leg, just above the ankle, and applied resistance. The subject tried to extend his or her knee, against the resistance of the observer, keeping the upper leg in the same position.
- **BF-ST**: The same initial setup as RF-VL. The observer placed a hand on the posterior side of lower leg, just above the ankle, and applied resistance.
- **AM**: One foot was lifted off the ground. The knee was fully extended and the observer placed hands just above the knee, on the medial side of the leg. The subject tried to pull his or her leg medially to the other leg whilst the observer exerted lateral resistance.
- **Gmed**: One foot was lifted off the ground. The knee was fully extended and the observer placed hands just above the knee, on the lateral side of the leg. The subject tried to push his or her leg laterally to the other leg whilst the observer exerted resistance.
- **Gmax**: One foot was lifted off the ground and the knee was fully extended. The observer placed his or her hands just below the knee, on the anterior side of the lower leg. The subject performed hip extension against the resistance.
- **TA**: The hip and knee were flexed to approximately 90 degrees. The observer places its hands on top of the toes. The subject performed dorsiflexion.
- **GM**: Subject flexes its hip and knee to approximately 90 degrees. The observer places its hands under the toes. The subject performed plantar flexion.

It is important to keep in mind that no standard exist for performing MVCs^20^ and many possible ways of normalizing exist. In hindsight these MVCs could have been performed differently, preferably while the subject was seated or laying down. sEMG in this work was normalized using the peak dynamic method, similar to Bovi *et al*.^5^. The advantage of this method is that it does not require additional procedures and could also be used for non-able-bodied subjects.

## Data Records

Data are stored in the 4TU repository available at 10.4121/20418720^21^. For each subject a separate HDF5 file^22^ was created. These files contain the measurement moment named ‘Day_X’ with X the number of the measurement moment. Inside these measurement moments there are files called ‘Trial_YY’, with YY the trial number, containing the different data types and ‘MVC’ containing the sEMG maximum voluntary contractions of each measurement moment. Note that only MyPredict 3 contains multiple measurement moments per subject.

The different data types are acceleration (Acc), angular velocity (Gyr), joint angles (Ang), Orientation (Ori) and electromyography (EMG). Inside each file there are trials containing data arrays with the corresponding data. Data arrays are named as follows: Type_Side_Loc. Type is one of the six data types, Loc is the location of the sensor and Side is the side of the location, either Left, Right or empty. For example Ang_Right_Knee contains the 3D joint angles of the knee, Gyr_Pelvis contains the 3D angular velocity of the pelvis IMU and EMG_Left_VL contains the sEMG data of the left vastus lateralis. Orientation is the orientation of the pelvis in space, expressed in Euler angles. Separate data types are ‘Labels’, which contains manual placed activity labels for each timestamp and ‘Time’ which indicates the timestamps per file. Marker data (Mrk) are stored in a separate group, ‘Markers’ with their own ‘Time’ array, as they have a different sample frequency (100 Hz) compared to the other data types (1000 Hz). An overview of all data types and locations is shown in Table 2.

**Table 2:** Overview of the various data types and locations of placement.

Type	Side	Location
Acc, Gyr	Left, Right	Thigh, Shank, Foot
Acc, Gyr		Pelvis, T8
Ang	Left, Right	Hip, Knee, Ankle
Ang		L5S1, Pelvis
EMG	Left, Right	RF, VL, ST, BF, GMax, TA, GM, Gmed (MP1), AM (MP3)
EMG	Right	MA (Multi-array grid, 4 × 16 (MP3))
		MA_f (Multi-array grid front RF/VL 4 × 8 (MP2))
		MA_b (Multi-array grid back BF/ST 4 × 8 (MP2))
Mrk	Left, Right	ASI, Acromion, FifthMetatarsal, FirstMetatarsal, GreaterTrochanter, HeelFoot
		KneeLatEpicondyle, KneeMedEpicondyle, LatMalleolus, MedMalleolus, Toe
Mrk		C7SpinalProcess, IJ, Sacrum, T12SpinalProcess, T4SpinalProcess
Label		
Time		

Each saved variable is named according to the structure Type_Side_Location. Exceptions are Label, containing the activity labels and Time, which contains an array of timestamps, to be used for file synchronization.

The metadata of the subjects as shown in Table 1 is stored in the HDF5 files as well. These are height, weight and age of the subject during the measurement.

## Technical Validation

**Fig. 6:**
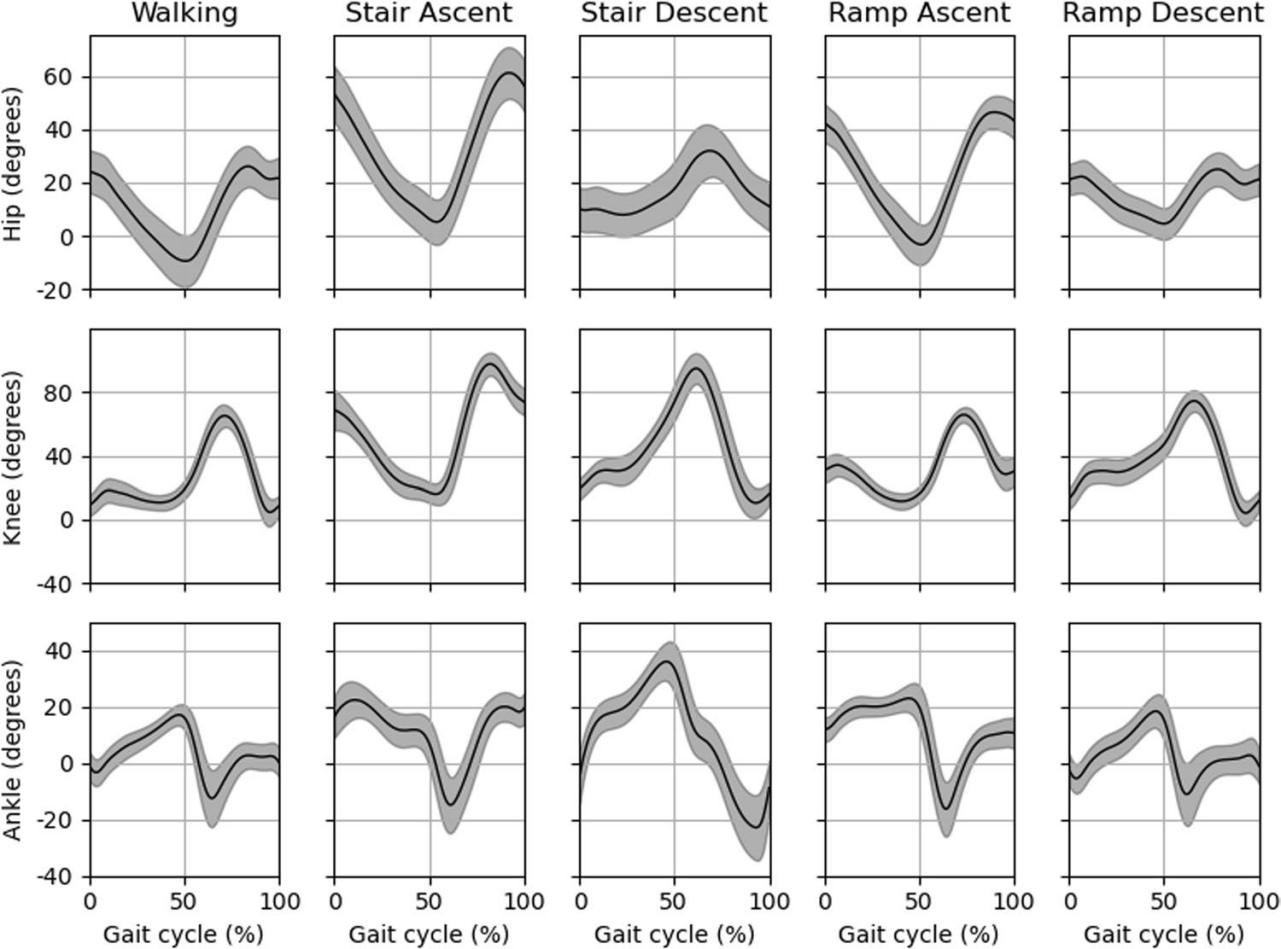
Average joint angles of the hip, knee and ankle in sagittal plane of all subjects during walking, stair ascent, stair descent, ramp ascent and ramp descent. Gait cycles are from initial contact to initial contact.

**Fig. 7:**
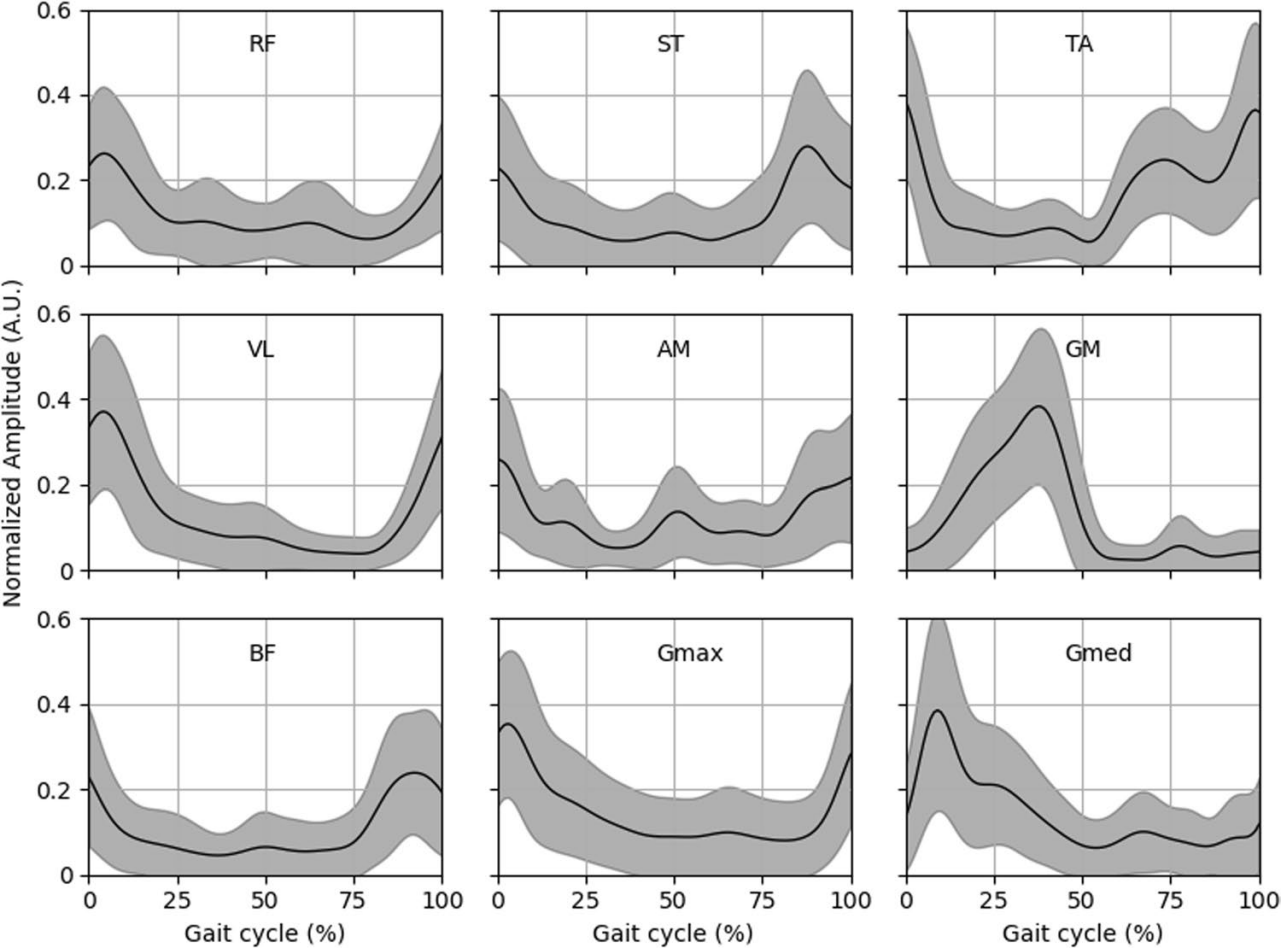
Average sEMG profiles during overground walking of the bipolar sEMG collected in MyPredict 1 and MyPredict 3. sEMG is normalized using the peak dynamic method over all strides per subject. Gait cycles are from initial contact to initial contact. Gmed was only measured in MyPredict 1 and the AM was only measured in MyPredict 3.

To validate the quality of our data sets we investigated the gait cycle averages for the gait-related activities, as shown in Fig. 6 for joint angles and in Fig. 7 for sEMG. Initial contacts were determined using the sagittal angular velocity of the shank, as described by Maqbool *et al*.^23^. Hip flexion, knee flexion and ankle dorsiflexion are defined as a positive value in Fig. 6. sEMG was normalized using the peak dynamic method^24^, similar to Bovi *et al*.^5^. Per measurement session the maximum activation during overground walking was determined and this value was used for normalization.

### Comparison with other data sets

**Fig. 8:**
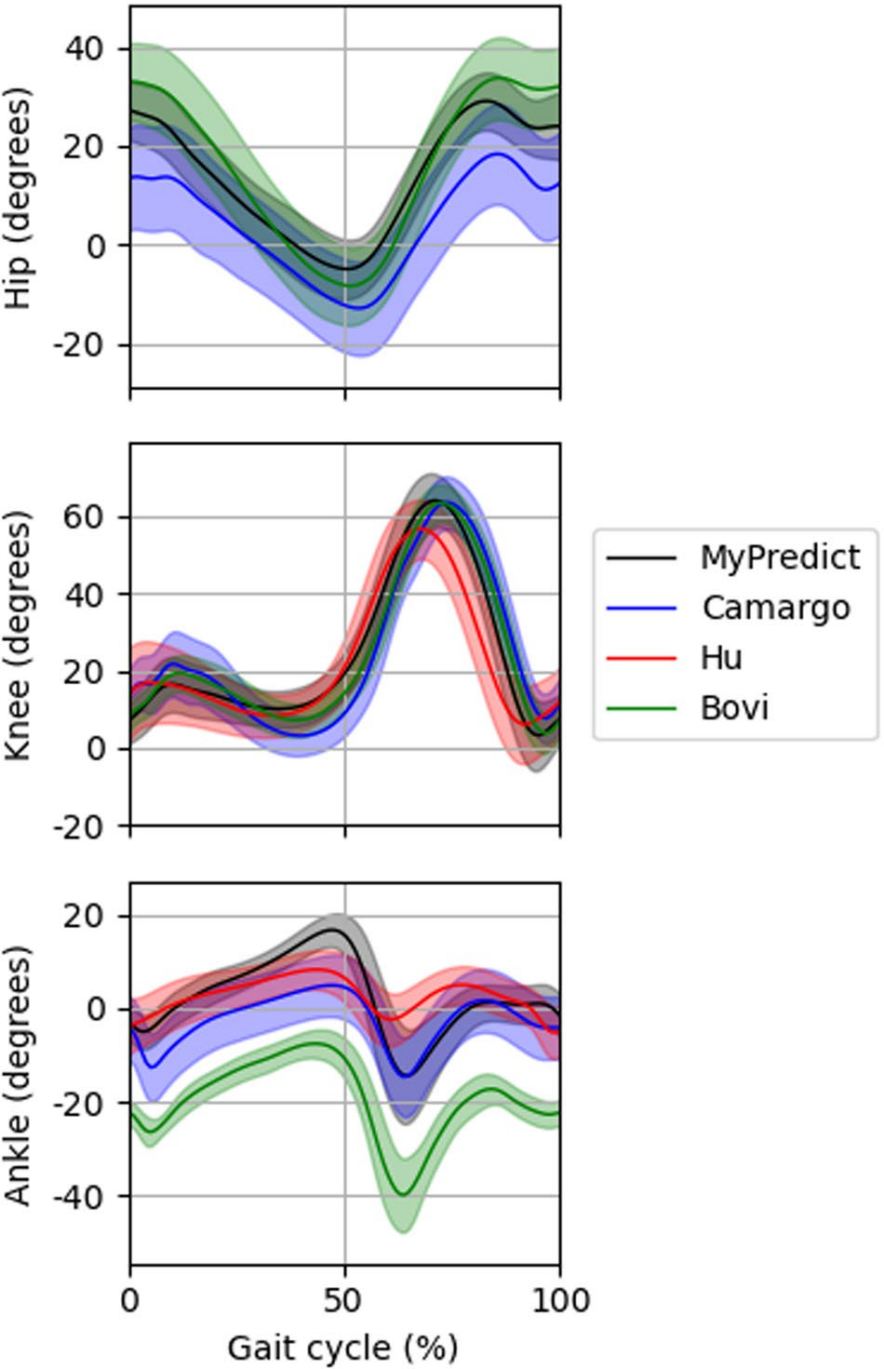
Hip, knee and ankle angles in the sagittal plane during overground walking compared with the data collected by Camargo *et al*.^7^, Hu *et al*.^6^. and Bovi *et al*.^5^.

We compared our data with the data set described by Bovi *et al*.^5^, Camargo *et al*.^7^ and Hu *et al*.^6^. Kinematics were measured using the MVN Link suit by Xsens (Enschede, The Netherlands). Xsens have shown to be capable of measuring human kinematics with excellent correlation compared with optical motion trackers^13,25^. In Fig. 8 average joint angles are shown for overground walking of the different data sets. No data was collected by Hu *et al*.^6^ for the hip joint. Correlation coefficients for the hip flexion/extension angle were 0.99 with Bovi *et al*.^5^ and 0.98 with Camargo *et al*.^7^. For knee flexion/extension angle the correlation coefficients were 0.99, 0.94, 0.95 compared with Bovi *et al*.^5^, Camargo *et al*.^7^ and Hu *et al*.^6^ respectively. For ankle plantar/dorsiflexion the correlation coefficients were 0.96, 0.88 and 0.72 compared with Bovi *et al*.^5^, Camargo *et al*.^7^ and Hu *et al*.^6^ respectively. Note that there is a offset between the ankle angle measured by Bovi *et al*.^5^ due to a different definition for the ankle angle. The joint angles in this work show excellent correlation with joint angles measured using an optical motion tracking system. Next to that, the hip and knee angle show excellent correlation compared with wearable motion tracking and strong correlation for the ankle.

For sEMG the correlation coefficient per muscle can be found in Table 3. It can be seen that sEMG shows excellent correlation with the online available data sets as well. These correlations were 0.86–0.95 compared with Bovi *et al*.^5^, 0.72–0.98 compared with Camargo *et al*.^7^ and 0.82–0.96 compared with Hu *et al*.^6^.

**Table 3:** Correlation coefficients for sEMG in the MyPredict data set compared with the data collected by Camargo *et al*.^7^, Hu *et al*.^6^ and Bovi *et al*.^5^ during overground walking.

Data set	Gmax	Gmed	RF	VL	BF	ST	AM	TA	GM
Camargo *et al*.^7^		0.72	0.97	0.95	0.89	0.88		0.94	0.98
Hu *et al*.^6^			0.94	0.93	0.95	0.96		0.82	0.94
Bovi *et al*.^5^	0.88		0.93	0.95*	0.95			0.86	0.98

Not each online data set measured the same muscles, so certain comparisons have not been made. *NB: The data set by Bovi *et al*.^5^ did not contain data of the vastus lateralis, so we compared sEMG from the vastus lateralis from the MyPredict data set with the sEMG of the vastus medialis measured by Bovi *et al*.^5^ as it can be expected that the vastus lateralis and medialis will have similar activity during overground walking.

### Limitations

Although this data set contains data to investigate differences between EMG types and activity recognition strategies, it should be kept in mind that the data was collected only with able-bodied young individuals. As Bovi *et al*.^5^ showed, there are kinematic differences between young and older adults. Another limitation is that kinematics were collected an IMU-based motion capture system, which is not considered to be the golden standard for motion tracking. Optical motion tracking in the lab is regarded as the most reliable way of measuring kinematics, although studies have shown that IMU tracking can be as accurate as optical motion tracking^13,25^. In our technical validation we have also showed excellent correlation with measurements conducted with optical motion capture systems. Next to that, the major advantage of using an IMU-based motion capture system is the possibility to measure subjects while transitioning freely from one activity to the next and also outside of the lab, which were for us some of the main reasons to use an IMU-based motion capture system. Another limitation of this study is the absence of force related data, such as data from force plates. This data set can therefore not be used to estimate full-body kinetics during gait-related activities.

## Data Availability

The scripts that facilitate re-use of the data can be found in the GitHub repository https://github.com/Rvs94/MyPredict. These scripts were developed and written in Python 3.9. All required software packages are open-source and available online.
